# Jejunal Diverticular Perforation Causing Small Bowel Obstruction in a Type 4 Hiatal Hernia: A Rare Case Report of a Nonagenarian Patient and Review of Relevant Literature

**DOI:** 10.1155/2017/8412927

**Published:** 2017-10-10

**Authors:** Saptarshi Biswas, Shekhar Gogna, Prem Patel

**Affiliations:** ^1^Department of Trauma and Acute Care Surgery, Allegheny Health Network, Pittsburgh, PA, USA; ^2^Department of General Surgery, Westchester University Medical Center, Valhalla, NY, USA

## Abstract

Type IV paraesophageal hernia (PEH) is very rare and is characterized by the intrathoracic herniation of the abdominal viscera other than the stomach into the chest. We describe a case of a 90-year-old male patient who presented at our emergency department complaining of epigastric pain that he had experienced over the past few hours and getting progressively worse. On the day after admission, his pain became severe. Chest radiography revealed an intrathoracic intestinal gas bubble; emergency exploratory laparotomy identified a type IV PEH with herniation of only the jejunum with perforated diverticula on mesenteric side through a hiatal defect into mediastinum. There are a few published cases of small bowel herniation into the thoracic cavity in the literature. Our patient represents a rare case of an individual diagnosed with type IV PEH with herniation of jejunum with perforated diverticula.

## 1. Introduction

Hiatal hernia is classified into four types; about 95% are type I or sliding hernias, whereas less than 5% are paraesophageal hernias (PEH) (types II–IV). Type IV PEH is itself very rare in the group [1]. Type IV hiatal hernia is defined as herniation of other abdominal viscera apart from stomach. Literature reports herniation of colon, ileum, jejunum, pancreas spleen, or greater omentum. Diverticular disease of the small bowel is a rare entity and often is an incidental finding on imaging, endoscopy, or laparoscopy [2]. Jejunal diverticula is the least common type with an incidence of about 1 percent [3]. Often asymptomatic, they may cause complications requiring surgical intervention in about 10 to 30 percent of the patients [4]. It has been a general teaching in surgery that rarely are two separate diagnoses evoked to explain a single symptom. However, this case demonstrates the presence of two separate but related diagnoses: perforated jejunal diverticula and a type IV hiatal hernia.

## 2. Case Report

A 90-year-old male presented to the emergency department with complaints of midepigastric and right upper abdominal pain. According to the patient the pain started the same day; it was a rapidly developing discomfort which he described as an “aching sensation.” There was associated nausea with 2-3 bouts of vomiting. He denied fever or chills. He did move his bowel twice since the pain started. Past surgical history was significant for history of right hemicolectomy for carcinoma colon, sigmoid colon resection for diverticulitis, laparoscopic cholecystectomy, appendectomy, cataract, and history of small bowel obstruction leading to small bowel resection 2 years back. Physical examination revealed an elderly white male patient. Vitals include temperature of 96.3 F, pulse rate of 87, respiratory rate of 27, and blood pressure on presentation of 194/83.

The abdomen was grossly doughy and mildly distended. However on palpation there was diffuse tenderness across the abdomen, especially in the right upper quadrant with guarding but no definite rebound.

Serum biochemistry was within normal limits.

Preoperative imaging consisted of X-ray abdomen followed by the CT scan.

**Figure 1:**
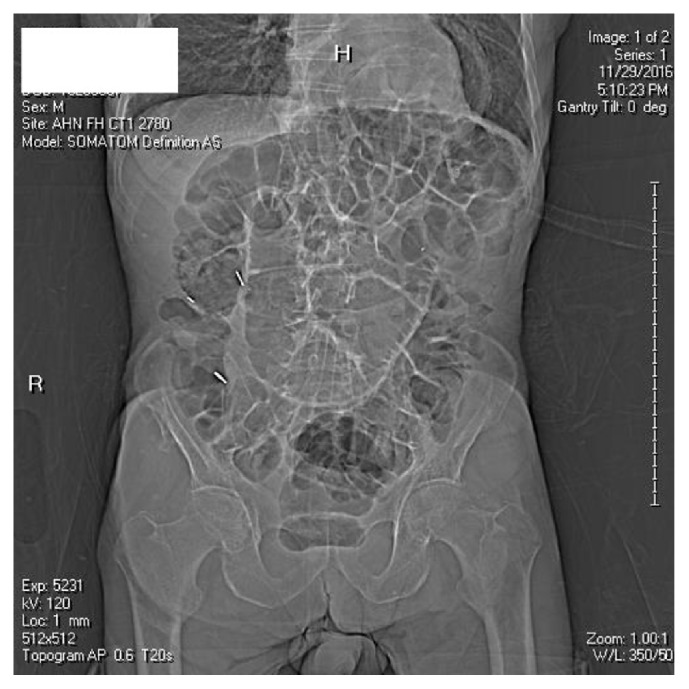
Abdominal X-ray.

A plain film abdomen (Figure 1) showed diffuse gaseous distention of bowel with loops of dilated small bowel measuring up to 6.5 cm in diameter. A large hiatal hernia distended with gas containing the stomach along with possible bowel protruding into the hernia was also seen.

**Figure 2:**
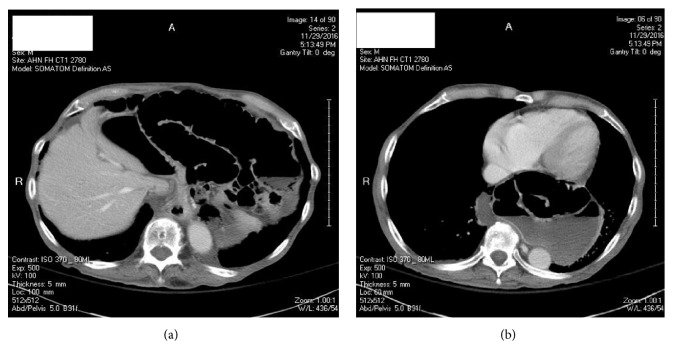
CT scan.

The CT scan (Figures 2(a) and 2(b)) showed large type IV hiatus hernia, which was distended by fluid and gas. Based on the new finding of air-fluid level containing fluid crescent communicating with the general peritoneal cavity, there was suspicion of bowel perforation relating to either the hiatus hernia or the small bowel obstruction.

Based on physical examination and imaging, decision was made to take up the patient for surgery. Generous midline laparotomy incision was made; due to prior surgeries and significant adhesions, extensive lysis of adhesions was done. All herniated small bowel and stomach protruding through the hiatus were reduced back into the peritoneum. The perforation was identified in proximal diverticula of the jejunum. Due to limited length available and advanced age of the patient, wedge resection of the diverticulum was performed and it oversewn in two layers. Hiatus was repaired with interrupted figure of "eight" stitches using #0 Prolene suture. No mesh was placed because of perforated viscus. The patient tolerated the procedure well. He was started on clears on postoperative day 4 which he tolerated and gradually advanced to regular diet. The patient was discharged on postoperative day 7.

## 3. Discussion

Henry Ingersoll Bowditch coined the term paraesophageal hiatus hernia (PHH) in 1853 [5]. As described, type IV paraesophageal hernia is characterized by the displacement of the stomach plus other organs such as the colon, spleen, and small bowel into the chest. Our patient had associated perforated jejunal diverticula. Jejunal diverticulosis is usually asymptomatic, and uncomplicated jejunal diverticulosis does not need any surgical intervention. Acute presentations of this pathology are gastrointestinal bleeding, intra-abdominal abscess, perforation with diffuse peritonitis, small bowel volvulus, and jejunocolic fistula [6]. Chronically, it can present as malabsorption syndrome [7]. In contrast to Meckel's diverticulum, which is a true diverticulum presenting at the antimesenteric border, jejunal diverticula are false diverticula and present usually along the mesenteric border of the small bowel [8]. Our patient had jejunum in the posterior mediastinum which had herniated thorough diaphragmatic hiatus. There are about 6 cases of type IV PEH till date reporting ileum as content, with jejunum having never been reported as content in PEH. There are two potential mechanisms of pathogenesis of PEH; with increasing age there is laxity in crura of diaphragmatic hiatus which allows herniation and, secondly, there is a large defect in phrenoesophageal membrane which allows other organs to herniate [9]. Clinical features of type IV PEH are usually divided into three types:

(1) Asymptomatic in which PEH is discovered while doing imaging for unrelated cause

(2) Nonspecific, including acid reflux, upper epigastric pain, heart burn, vomiting, and symptoms of aspiration or iron deficiency anemia due to Cameron lesions in herniated stomach.

(3) Patients present with features of frank obstruction or incarceration of involved bowel and sepsis (Figures 2(a) and 2(b)) [10].

In patients who develop severe epigastric pain, one must suspect incarceration and/or obstruction [11].

Features of small bowel obstruction in diverticular disease are secondary to wall edema and peripheral inflammation and mesenteric lymphadenopathy [12]. Obstruction in diverticula leads to perforation due to weakness in diverticular wall.

Our patient had evidence of type IV hiatal hernia on one of the CT scans performed unrelated to this hospital admission. The presence of severe epigastric tenderness, chest pain, and dyspnea are grounds for considering a differential diagnosis of type IV PEH. Our patient demonstrated progression from nonspecific features to symptoms of obstruction and incarceration. The nature of progression of symptoms whether static or evolving while taking clinical history points towards importance of early intervention. Diagnosis of PEH is usually via X-ray and CT scan. On plain X-ray a hiatal hernia is seen as a rounded soft-tissue opacity or an air-filled hyperlucency in the retrocardiac region, with or without an air-fluid level [13]. CT scan defines the anatomy of hiatus and contents in mediastinum. Surgery is the only way to restore herniated organs back into the abdominal cavity, repair the hiatus to prevent future recurrence, and treat the herniated viscera accordingly. Surgery is elective or emergent; it can be both laparoscopic and open. Guidelines recommend laparoscopic repair of PEH in elective setting.

Acute complications in PEH are surgical emergency; both laparoscopic and open approach have been used and debate also exists as to which approach is preferable. Patients undergoing emergent surgery for hiatal hernia repair have more adverse prognostic factors and more major complications (38 versus 18%; *p* < 0.001) and deaths (8 versus 1%; *p* < 0.001) [14].

Some reports suggest that the laparoscopic repair results in higher recurrence rates compared with open surgery [15]. In reality the type of surgical approach should be based on general health of patient and acuity of condition. Principal of surgery remains the same, that is, reduction of contents back into cavity, ensuring its viability, followed by resection or repair of the involved gut along with repair of the hiatus.

## 4. Conclusion

Hiatus hernia is liable to the same complications as hernias elsewhere. In cases of past surgical history, the diagnosis of these hernias is challenging and can be easily missed or delayed in the emergency setting due to the nonspecific symptoms and their low incidence. Surgery offers the definitive chance of cure. After ruling out all causes of SBO/perforation, the index of suspicion should remain on such rare pathology. The early diagnosis and the immediate laparotomy mediated the successful management and an uneventful postoperative course.
